# Fibroblast activation protein and the tumour microenvironment: challenges and therapeutic opportunities

**DOI:** 10.3389/or.2025.1617487

**Published:** 2025-07-16

**Authors:** Hsing Hwa Lee, Zeyad Al-Ogaili

**Affiliations:** ^1^Department of Medical Oncology, Fiona Stanley Hospital, Perth, WA, Australia; ^2^Department of Nucelar Medicine, Fiona Stanley Hospital, Perth, WA, Australia

**Keywords:** fibroblast activation protein (FAP), cancer associate fibroblasts (CAFs), tumour microenvironment (TME), immunotherapy, cancer

## Abstract

Fibroblast Activation Protein (FAP) has emerged as a critical player in cancer biology, particularly in shaping the tumour microenvironment (TME) and influencing immunotherapy outcomes. FAP-positive cancer-associated fibroblasts (CAFs) play multiple roles in tumour progression and immune modulation. FAP, predominantly expressed on CAFs, contributes significantly to extracellular matrix remodelling, angiogenesis, and the creation of an immunosuppressive milieu. There are complex interactions between FAP-positive CAFs and various components of the immune system, highlighting their impact on T cell function and macrophage polarisation. This makes FAP a promising target for cancer therapy and potentially as a biomarker for immunotherapy treatment response. This review highlights the clinical challenges to target FAP and also addresses the heterogeneity of CAFs with the need for more refined characterisation to enhance therapeutic strategies and future research directions.

## 1 Introduction

Immunotherapy has significantly transformed the landscape of cancer treatment by introducing a range of innovative strategies, including checkpoint inhibitors, CAR T-cell therapies, and personalised cancer vaccines. These advances in immunotherapy have shown considerable promise in improving patient outcomes but also face notable limitations due to their varying efficacy across different cancer types and among diverse patient populations. The variation in treatment response, coupled with the high costs associated with these therapies and the potential for severe, sometimes life-threatening side effects, underscore the critical need for accurate and reliable prediction of immunotherapeutic outcomes. This requirement for precision in forecasting responses is essential not only to enhance therapeutic efficacy but also to minimise adverse effects and optimise patient care in oncology (1, 2).

Recent advances in the field of oncology have significantly deepened our understanding of the tumour microenvironment (TME) and its crucial role in modulating responses to various therapies, including immunotherapy. The inherent complexity of the TME, shaped by its diverse cellular and molecular constituents, plays a pivotal role in influencing the efficacy of immunotherapies, either by promoting or inhibiting immune evasion and tumour growth. Among the most critical elements within the TME are the cancer-associated fibroblasts (CAFs). Predominantly prevalent in solid tumours, CAFs can make up to 90% of the cellular mass in certain cancers. They are known for their ability to secrete a range of cytokines and growth factors that can significantly reshape the landscape of immune surveillance and alter the overall responses to therapy, making them attractive targets in the development of new therapeutic strategies (3, 4). Fibroblast Activation Protein (FAP), a pivotal marker and mediator expressed by CAFs, has emerged as a significant diagnostic, therapeutic, and prognostic target due to its multifaceted roles in the tumour microenvironment. This review focusses on opportunities in targeting FAP and the unique challenges in the TME especially with immunotherapy treatment.

## 2 Cancer-associated fibroblasts (CAFs)

### 2.1 Origin and heterogeneity

Cancer-associated fibroblasts (CAFs) are pivotal elements within the tumour microenvironment, originating from diverse sources that contribute to their significant heterogeneity. These sources include local activation of resident fibroblasts, recruitment of bone marrow-derived mesenchymal stem cells, and transitions from epithelial and endothelial cells through processes known as epithelial-mesenchymal transition (EMT) and endothelial-mesenchymal transition (EndMT) (5–7). These varied origins contribute to the spectrum of functional capabilities of CAFs observed across different tumour types and individual cancers.

The heterogeneity of CAFs is further delineated by their expression of specific markers, which vary based on their origin and the local tumour environment. Common markers include alpha-smooth muscle actin (α-SMA), fibroblast activation protein (FAP), and vimentin, which are indicative of their activated state and mesenchymal origin, aiding in distinguishing CAFs from normal fibroblasts. Additionally, more specific markers like S100A4 and PDGFRβ have been identified, helping to classify CAFs into subpopulations such as myofibroblastic CAFs and inflammatory CAFs, each associated with distinct functions within the tumour stroma, contributing variably to cancer progression and response to therapy (8, 9).

### 2.2 Functions in the tumour microenvironment

CAFs shape the tumour microenvironment by remodelling the extracellular matrix (ECM), supporting angiogenesis, and modulating immune responses. They secrete ECM components and matrix metalloproteinases (MMPs), which restructure the tumour stroma, increasing stiffness and invasiveness. This remodelling promotes tumour growth and invasion while also enhancing angiogenesis through VEGF release, sustaining the tumour’s nutrient and oxygen supply (5, 10)

A major function of CAFs is immune modulation. They release cytokines like TGF-β and IL-6, suppressing effector T cells and encouraging regulatory T cell (Treg) expansion. CAFs also produce chemokines that attract immunosuppressive cells such as myeloid-derived suppressor cells (MDSCs) and Tregs, creating an environment that allows tumour cells to thrive and evade immune detection. This immunosuppressive role significantly affects the success of immunotherapies (6–8, 11).

Interestingly, some CAFs secrete decorin, a protein that inhibits tumour growth and metastasis. This dual role reflects the complex, context-dependent nature of CAFs in tumour biology (10).

**FIGURE 1:**
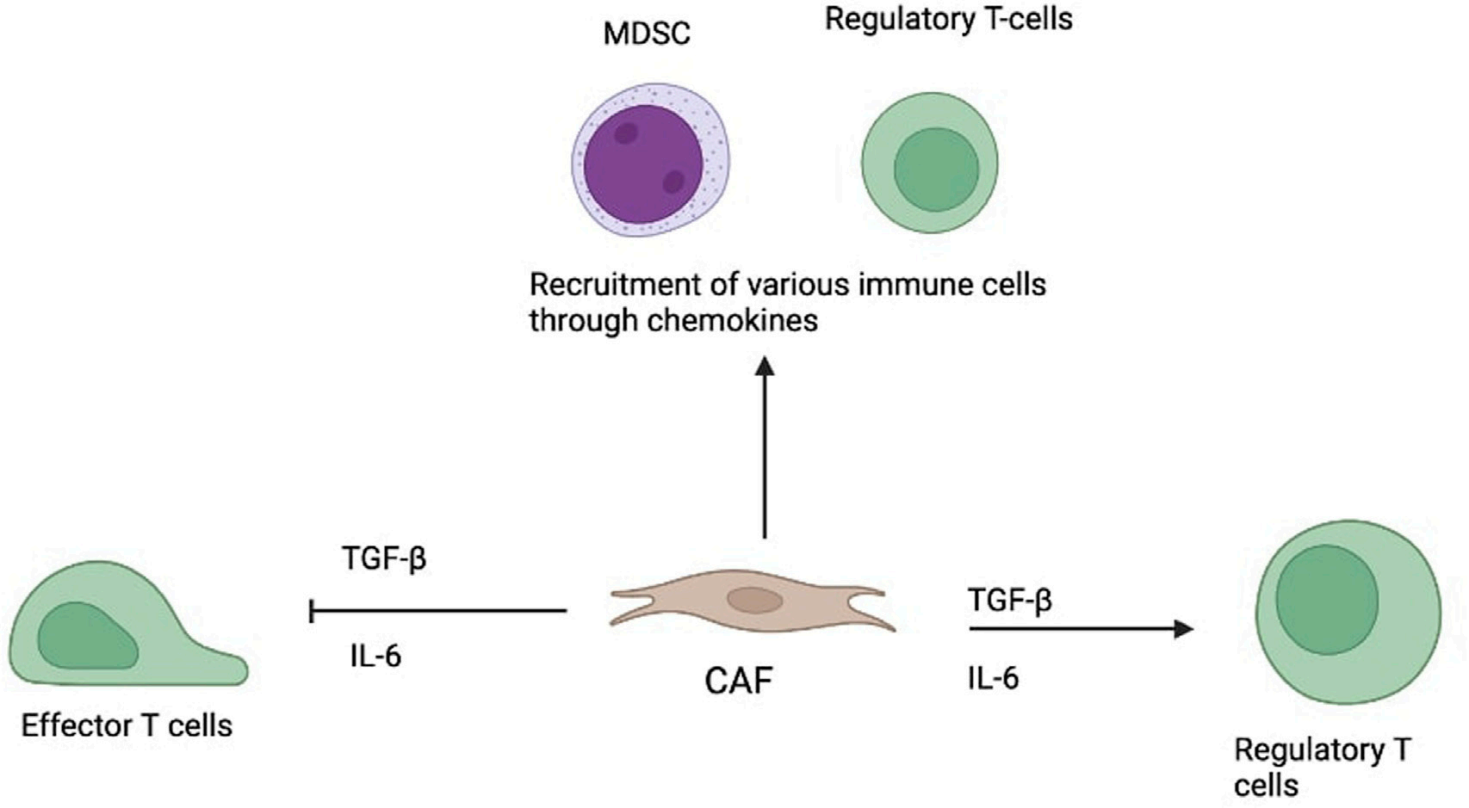
Role of CAF in modulating immune responses in the tumour microenvironment.

Given their multifaceted influence (see Figure 1), CAFs present promising targets for cancer therapy—particularly in boosting immunotherapy effectiveness by disrupting their tumour-supportive functions (5–7).

### 2.3 History and structural details of fibroblast activation protein

Fibroblast activation protein (FAP) was first identified by Rettig et al in the mid-1980s while studying cell surface antigens to characterise activated fibroblasts (12). They used a monoclonal antibody called F19, which detected an antigen on various cell types, including epithelial cancer cells, soft tissue sarcomas, granulation tissue in wound healing and foetal mesenchymal fibroblasts. This antigen was named “FAP” due to its strong expression on activated fibroblasts but not on normal fibroblasts or epithelial tumours (13).

The protein structure of FAP includes several key domains, namely, the large extracellular domain, transmembrane domain and a short cytoplasmic tail. The extracellular domain contains the catalytic alpha/beta-hydrolase domain, which houses the catalytic domain and the eight-bladed beta-propeller domain, which is important for the protein’s structure and function (13, 14). Within the catalytic domain, FAP possesses a catalytic triad typical of serine proteases, consisting of Serine (S624), Aspartate (D702) and Histidine (H734). This triad is crucial for its enzymatic activities, including both its dipeptidyl peptidase and endopeptidase functions. These serine proteases work together to catalyse the hydrolysis of peptide bonds. The serine residue in FAP’s active site acts as a nucleophile, enabling the cleavage of N-terminal Pro-X peptide bonds, whereX represents any amino acid except proline or hydroxyproline (13).

#### 2.3.1 Substrates of fibroblast activation protein

FAP exerts its effects through both dipeptidyl peptidase and unique endopeptidase activities, particularly targeting collagen types I and III after initial breakdown by matrix metalloproteases. This highlights its role in tissue remodelling and fibrosis (8, 9).

FAP also cleaves α2-antiplasmin (α2-AP), enhancing its inhibition of plasmin and slowing fibrinolysis, which promotes scar formation. The cleaved α2-AP binds fibrin 13 times faster, earning FAP the name antiplasmin-cleaving enzyme (APCE) (9, 12).

Additionally, FAP inactivates Fibroblast Growth Factor 21 (FGF21), a hormone vital for regulating glucose, lipid, and energy metabolism, and for protecting cells from inflammation and immunometabolic stress (9, 13).

#### 2.3.2 Enzymatic activity of fibroblast activation protein

Fibroblast activation protein (FAP) exhibits dual enzymatic activity: dipeptidyl peptidase and endopeptidase. The dipeptidyl peptidase activity cleaves Pro-X bonds at the N-terminus of substrates, such as neuropeptide Y and brain natriuretic peptide, thereby influencing neuropeptide signalling and cardiovascular regulation. Endopeptidase activity, unique to FAP, cleaves Gly-Pro-X sequences in denatured proteins such as collagen types I and III, aiding extracellular matrix (ECM) remodelling, tumour invasion, and fibrosis. FAP also enhances α2-antiplasmin fibrinolysis inhibition and inactivates fibroblast growth factor 21 (FGF21), thereby impacting metabolism and immune regulation. These enzymatic functions highlight FAP’s critical role in ECM remodelling and disease progression, establishing its potential as a therapeutic target (15, 16).

### 2.4 Fibroblast activation protein expression in normal tissue, benign and malignant pathology

#### 2.4.1 Fibroblast activation protein expression in normal tissues and benign disease

In healthy adult tissues, FAP expression is minimal or absent in organs like the uterus, cervix, placenta, breast, and skin. However, FAP can be selectively expressed during tissue remodelling in conditions like wound healing, embryogenesis, inflammation, and fibrosis (17).

FAP is upregulated on activated fibroblasts during wound healing. Keloid scars contain more FAP-positive fibroblasts than normal skin (18). In liver fibrosis, FAP is prominently expressed on hepatic stellate cells (HSCs), particularly in fibrotic septa near inflammation. These FAP-positive HSCs—typically α-SMA-negative—are thought to represent a fibrosis-driving subpopulation and serve as a stronger marker than GFAP (19, 20).

In Crohn’s disease, FAP is significantly upregulated in myofibroblasts within intestinal strictures—an effect not seen in ulcerative colitis. Immunohistochemistry and imaging confirm FAP activity in fibrostenotic regions (21, 22).

Arthritis also shows FAP upregulation. In osteoarthritis, chondrocyte surface FAP is elevated, with FAPI PET-CT scans demonstrating uptake in affected joints (21, 23).

Cardiovascular conditions like atherosclerosis and myocardial infarction show FAP expression in aortic smooth muscle cells and peri-infarct zones. FAP imaging can also detect early chemotherapy-induced myocardial injury (21).

IgG4-related disease, a fibrotic condition, demonstrates broad FAP expression, with imaging detecting more sites than symptoms suggest. Some benign tumours, like angiomyolipoma and solitary fibrous tumours, also show low-level FAP uptake compared to malignant lesions (21).

#### 2.4.2 Fibroblast activation protein expression in malignant disease

FAP is expressed in many cancers and contributes to tumour progression and metastasis. It is found on CAFs within the tumour stroma and, in some cases, on tumour cells themselves. FAP is commonly seen on fibroblasts surrounding epithelial cancers such as those of the skin, breast, prostate, colon, pancreas, and in sarcomas (24). Tumour cells expressing FAP include pancreatic adenocarcinoma, sarcoma, oesophageal and gastric cancers, colorectal cancer, mesothelioma, breast ductal adenocarcinoma, oral squamous cell carcinoma, glioma, ovarian, and cervical cancers (25). This specific localisation in both stroma and tumour cells makes FAP a promising prognostic and therapeutic target.

### 2.5 Functions of fibroblast activation protein in tumour biology

#### 2.5.1 Role in tumour invasion, metastasis, and immune evasion

FAP has been shown to promote tumour invasion through several mechanisms. FAP is associated with α3β1 integrin which allows FAP to localise to invadopodia and enhance extracellular matrix degradation and invasion. Studies with ovarian cancer cells showed that the inhibition of α3β1 integrin reduced FAP-induced proliferation and migration (15, 26). Besides that, FAP can directly affect cell motility and migration via its enzymatic activity with the PTEN/PI3K/Akt and Ras-ERK pathway (27).

FAP plays a vital role in angiogenesis by contributing to the reorganisation of ECM which helps to promote endothelial cell invasion and capillary growth (15). Studies involving gastric cancer biopsies showed increased micro-vessel density with cancers of higher FAP expression (28). FAP is localised around the invadopodia of endothelial cells and the endothelial cells of developing microvascular systems in multiple malignancies (15).

Moreover, FAP plays a significant role in mediating immune evasion within the TME. FAP is expressed by CAFs which contribute to immune suppression in the TME directly by promoting regulatory T cells (Tregs) and tumour-associated macrophages via secreted cytokines and indirectly by ECM remodelling and creating a physical barrier (29, 30). Pancreatic cancer mouse models depleted of CAFs showed improved efficacy of checkpoint inhibitor therapy, confirming the role of CAFs in TME immune suppression. The chemokine (C-X-C motif) ligand 12 (CXCL12) was suggested to be responsible for this process and is produced by FAP-positive CAFs (31). Another study suggested FAP expressing macrophages induces immunosuppression by releasing heme oxygenase-1, which creates carbon monoxide, which suppresses the pro-apoptotic effects of TNFα on endothelial cells (15, 32).

### 2.6 Cancer associated fibroblasts, fibroblast activation protein and the immune microenvironment

#### 2.6.1 Immune modulation by cancer associated fibroblasts

FAP positive (FAP+) CAFs have a substantial impact on the tumour immune microenvironment, influencing the behaviour and efficacy of immune cells in several mechanism.

1. Cytokine secretion: FAP + CAFs contribute to the creation of an immunosuppressive tumour microenvironment. They can inhibit the activity and proliferation of T cells, which are crucial for the immune response against tumours. This effect is often mediated through the secretion of immunosuppressive cytokines such as interleukin-6 (IL-6) and transforming growth factor-beta (TGF-β) (33–35).
2. Chemokine Secretion: FAP + CAFs secrete various chemokines that critically alter the recruitment and distribution of immune cells within the tumour microenvironment. For example, FAP + CAFs produce CXCL12, which has a dual role in attracting stromal cells and excluding effector T cells from tumour sites, thereby facilitating an immunosuppressive environment conducive to tumour growth. Additionally, CAFs can produce CCL2 (MCP-1), which attracts myeloid-derived suppressor cells (MDSCs) and macrophages that further support tumour growth and suppress anti-tumour immune responses (31, 36).
3. Modulation of Macrophages: FAP + CAFs influence macrophage polarization, promoting the differentiation of macrophages towards an M2-like phenotype. M2 macrophages are associated with tissue repair and tumour progression, as they produce anti-inflammatory cytokines and support angiogenesis and remodelling of the extracellular matrix (19, 37, 38).
4. Physical Barrier Formation: By remodelling the extracellular matrix, FAP + CAFs can physically impede the penetration of effector immune cells, such as cytotoxic T lymphocytes, into the tumour mass. This dense extracellular matrix can act as a physical barrier that limits the accessibility of immune cells to cancer cells (39–41).
5. Interaction with Other Immune Checkpoints: FAP + CAFs can affect the expression of other immune checkpoints on the surface of tumour cells or immune cells. For instance, they can promote the expression of PD-L1 on tumour cells, which interacts with PD-1 on T cells to inhibit their activation and function (42–44).
6. Modulation of Antigen Presentation by CAFs: CAFs play a significant role in regulating antigen presentation within the tumour microenvironment. These stromal cells can directly interact with dendritic cells (DCs) and other antigen-presenting cells (APCs), or they can modulate these cells’ functions indirectly through the secretion of cytokines and growth factors. This interaction can either enhance or suppress the immune responses, depending on the signals and the context within the tumour stroma, thereby influencing both the initiation and the propagation of anti-cancer immune activity (45–47).

### 2.7 Impact of FAP positive cancer associated fibroblasts on immunotherapy

CAFs are a heterogeneous group with multiple subsets, each playing distinct roles in the TME. The CAF-S1 subset has been identified as particularly important in immunosuppression. CAF-S1 fibroblasts attract T lymphocytes to the tumour site, enhance the survival of CD4^+^CD25^+^ T cells, and facilitate their transformation into CD25+FOXP3+ regulatory T cells (Tregs). Furthermore, CAF-S1 fibroblasts augment the immunosuppressive capabilities of Tregs, enabling them to inhibit the proliferation of effector T cells more effectively. In contrast, CAF-S4 fibroblasts do not exhibit these immunosuppressive properties (48).

Current therapeutic strategies targeting CAF-associated pathways focus on modulating the immunosuppressive effects of these cells. However, given the heterogeneity of CAFs and their complex interactions within the TME, further research is needed to develop more targeted approaches that can selectively inhibit pro-tumorigenic CAF subsets while preserving the anti-tumour functions of other fibroblast populations.

Strategies targeting CAF-associated immunosuppression include the depletion of CAFs, restoration of their quiescent phenotype, inhibition of effector molecules, and ECM remodelling. Simlukafusp alfa is an immunocytokine that binds FAP on tumour-associated fibroblasts and enhances immune cell activity by increasing antibody-mediated cytotoxicity through PD-L1 checkpoint inhibition. It is currently being tested in combination with anti-PD-1 therapy in Phase II trials for advanced melanoma, renal cell carcinoma, and pancreatic ductal adenocarcinoma, showing promising *in vitro* and *in vivo* results. Talabostat, a FAP inhibitor, is under investigation in advanced solid tumours alongside anti-PD-1 therapy, aiming to modulate TME-associated immunosuppression.

## 3 Challenges and future directions

### 3.1 Understanding the heterogeneity of cancer associated fibroblasts

It is a major challenge to define CAFs and their sub-populations and delineate their specific functions in cancer tumorigenesis as they can originate from a variety of cells. The classification of CAF subtypes also varies depending on the specific type of cancer being studied. For example, CAF-N (normal) and CAF-D (divergent) were described in human oral squamous cell carcinoma (OSCC), CAF-A and CAF-B in colorectal tumours and four subtypes CAF-S1 to CAF-S4 in human breast adenocarcinomas. The more common and unified classification of CAF based on molecular features has been suggested with the following major subtypes: myofibroblastic CAF (myCAF), inflammatory CAF (iCAF), interferon-response CAF (ifnCAF), antigen-presenting CAF (apCAF), matrix CAF (mCAF), RGS5+ CAF and CAF-S1 to CAF-S5 (49–53). See summary Table 1.

**TABLE 1:** Summary of four major subtypes of CAF based on molecular features and classification.

Subtype	Key biomarkers	Functional role	References
myCAF	SMA, FAP, collagen	ECM remodeling, contractility	(49, 50)
iCAF	FAP, IL-6, CXCL12	Immunosuppression, inflammation	(49–52)
apCAF	MHC class II, CD74	Antigen presentation	(49–51)
ifnCAF	IFN-response genes (IFIT1, CXCL1)	Anti-tumor immunity	(49–51)
mCAF	ECM genes, ensheathing tumour nests	Restricts T cell invasion	(49–52)
RGS5 + CAF	RGS5, pericyte markers	Myofibroblast-like, vascular niche	(49–52)
CAF-S1 to S5	FAP, PDPN, SMA (varies by subset)	Adhesion, immunosuppression, invasion	(51, 53)

FAP has been thought to be a potential broad biomarker for CAF and been proven to play an important role in cancer growth. FAP positive CAFs are thought to be instrumental in the development of immunosuppressive TME and high expression of FAP has been associated with poorer prognosis in various cancers. Consequently, FAP has attracted significant attention as a potential focus for developing therapeutic interventions and identifying biomarkers.

Despite numerous studies and technological advances, identifying a single biomarker that can definitively identify all CAFs in each tumour has proven challenging. The heterogeneity of CAFs makes it difficult to identify a single biomarker for all subtypes, while overlapping markers with other cell types complicate precise identification. CAFs demonstrate remarkable plasticity, transitioning between different states during tumour progression, and their functions and phenotypes vary based on tumour type, stage, and microenvironmental cues. The proportion and characteristics of CAF subpopulations evolve over time as tumours progress, and different subtypes show varying spatial distributions within tumours, adding another layer of complexity (54, 55).

Cancer-associated fibroblasts (CAFs) are dynamic components of the tumour microenvironment (TME), playing a crucial role in coordinating interactions between cancer cells and host matrix responses. The TME contributes significantly to CAF heterogeneity, with diverse subpopulations emerging in response to various environmental factors (55). This plasticity allows CAFs to adapt their phenotypes to environmental cues. Notably, different CAF subsets exhibit distinct spatial distributions within the tumour, highlighting the microenvironment’s influence on their localisation (54). Furthermore, the microenvironment influences metabolic interactions between CAFs and cancer cells, impacting tumour progression. CAFs also play a role in extracellular matrix (ECM) remodelling, both responding to and shaping the TME (54).

Future research directions to address these challenges include utilising advanced single-cell analysis techniques, validating findings using complementary methodologies such as CyTOF, multiplex flow cytometry, and multiplex immunostaining, and functional validation using various *in vitro* and *in vivo* model systems to understand the biological significance of proposed CAF subpopulations. Establishing a standardised classification system, investigating the role of the tumour microenvironment in shaping CAF heterogeneity and function, exploring metabolic interactions between CAFs and cancer cells, and studying ECM remodelling will contribute to a more comprehensive understanding of CAF biology and its impact on tumour progression (54).

### 3.2 Optimizing fibroblast activation protein targeted therapies

#### 3.2.1 FAP as a biomarker for immunotherapy

FAP could be a potential biomarker to predict response to immunotherapy treatment. Higher FAP levels were found to correlate with poorer response and clinical outcomes in bladder urothelial carcinoma and cutaneous melanoma patients undergoing treatment with immune checkpoint inhibitors (56). On the other hand, pre-clinical mouse models with head and neck cancer show that absence of FAP positive CAF surprisingly did not affect tumour progression or sensitise tumours to combination cisplatin and anti-PD1 treatment. FAP positive CAF were also not found to increase tumour progression or recurrence in mouse models. It is possible that there may be a mismatch between gene and protein expression as FAP gene expression was negatively associated with outcomes (57). More studies are required to study and confirm FAP as a potential biomarker for immunotherapy response in various types of cancer.

#### 3.2.2 Challenges in FAP-targeted therapies

Despite initial interest in targeting FAP for cancer treatment, a phase II exploratory trial of monoclonal antibody sibrotuzumab targeting FAP in metastatic colorectal cancer was discontinued early as it did not show efficacy (58). Unsurprisingly, another trial with FAP inhibitor talabostat in metastatic colorectal cancer patients also proved ineffective. There were no objective responses seen in all 28 participants in the trial. Laboratory analysis showed significant but incomplete inhibition of FAP enzymatic activity in the blood (59). The failure of these trials may be attributed to the heterogeneous nature of cancer-associated fibroblasts (CAFs) and the function of fibroblast activation protein (FAP), which can promote tumorigenesis in certain tumours while inhibiting it in others. Therefore, targeted treatment alone against FAP may not be effective until we are able to delineate FAP subtypes accurately and perform personalised targeted treatment. However, with an improved understanding of the tumour microenvironment, current research focuses on combination therapies to optimize FAP-targeted approaches, particularly involving the immune system. These include combining FAP inhibitors with immunotherapy and developing FAP-targeted CAR-T cells to target cancer-associated fibroblasts. A phase II basket study combining talabostat and immune checkpoint inhibitor pembrolizumab also showed limited efficacy without any objective response (60). While FAP-CAR-T cells show promise in activating the immune system and eliminating target cells, concerns about on-target off-tumour toxicity due to low-level FAP expression in healthy tissues persist. Ongoing clinical trials are investigating FAP-CAR-T cells, both alone and in combination with immunotherapy or other targets like Nectin-4, to address these challenges and improve efficacy (59, 61).

#### 3.2.3 Advances in FAP theranostics

FAP is an attractive target for molecular imaging for cancer as it is minimally expressed in normal tissues hence is a perfect target for theranostics. Radionuclide therapy targeting FAP such as 177Lu-EB-FAPI is being investigated and the first-in-human trial for metastatic radioiodine-refractory thyroid cancer was conducted in 12 patients with objective response rate of 25% (62). This early phase study showed that this radioligand therapy is safe and paves the way for future radioligand studies. Newer strategies could investigate combining immunotherapy treatment with radiotherapy which could potentially increase infiltration by cytotoxic T-cells within the TME hence enhancing the potency of immunotherapy. Pre-clinical studies using LNC1004 is supportive of this strategy and is shown to upregulate tumour PD-L1 expression (63).

### 3.3 Personalized treatment approaches

FAP positive CAFs present both challenges and opportunities for targeted cancer therapies. Promising strategies have emerged, including FAP-activated prodrugs that selectively target tumour stroma, inhibition of specific signalling pathways involved in CAF-cancer cell crosstalk, and repurposing existing drugs like losartan for modulating the tumour microenvironment. Pre-clinical studies indicate that losartan, an angiotensin II receptor type 1 antagonist, demonstrates potential in reducing cancer-associated fibroblast (CAF) activity due to its anti-fibrotic properties (64, 65).

Further research is needed in several areas to advance CAF-targeted therapies. These include validating alternative biomarkers for CAF subtypes, understanding CAF-TME interactions across various cancer types, conducting longitudinal studies on CAF dynamics and subtype interconversion, exploring combination therapies with CAF-targeted approaches, and developing advanced 3D models to replicate complex tumour microenvironment interactions (66–68). Addressing these research gaps will be crucial for developing personalised therapy approaches that characterise CAF subtypes, comprehensively analysing the tumour microenvironment and focusing on tumour-supportive CAFs rather than broad depletion.

## 4 Conclusion

In conclusion, FAP-positive cancer-associated fibroblasts (CAFs) are pivotal in orchestrating the complex interplay within the tumour microenvironment that crucially influences cancer progression. Due to their heterogeneity, CAFs play diverse roles, from extracellular matrix remodelling and angiogenesis to intricately modulating the immune landscape. FAP, a salient marker of CAFs, is emerging as a critical diagnostic and therapeutic target. Its role in immune modulation is particularly compelling as it facilitates the creation of an immunosuppressive environment that can shield the tumour from immune surveillance. This makes FAP not only a target for traditional therapies but also a potential linchpin in combination with emerging immunotherapies.

However, harnessing the full potential of targeting FAP-positive CAFs faces several challenges. Key among these is the need for a more refined characterization of CAF subtypes to tailor therapies more precisely and to avoid the broad-brush effects that could inadvertently promote tumour progression. Moreover, the off-target effects of FAP-directed therapies necessitate cautious development to ensure safety and efficacy.

Future research should focus on advancing imaging techniques that can accurately identify and monitor FAP expression dynamically within the tumour milieu. Improving CAF classification systems will enhance our understanding of their roles and interactions within the tumour, guiding more effective combination therapies. By addressing these challenges, targeting FAP-positive CAFs holds the promise of crafting more nuanced and potent strategies in cancer therapy. Continued investigation into this field is essential and promises to substantially advance our capabilities in cancer treatment, particularly in the era of immunotherapy, ultimately improving patient outcomes and expanding the horizons of precision medicine.
